# Preoperative Risk Factors for Acute Postoperative Atrial Fibrillation in Patients Undergoing Mitral Valve Repair for Degenerative Mitral Regurgitation: Insights Into Cardiac Geometry

**DOI:** 10.31083/RCM38938

**Published:** 2025-08-29

**Authors:** Hang Xu, Xinhe Xu, Jiexu Ma, Shanshan Zheng, Wu Song, Zhaoji Zhong, Sheng Liu

**Affiliations:** ^1^Department of Cardiovascular Surgery, Fuwai Hospital, National Center for Cardiovascular Diseases, Chinese Academy of Medical Sciences and Peking Union Medical College, 100037 Beijing, China

**Keywords:** atrial fibrillation, mitral valve repair, echocardiography, risk factors, interventricular septum

## Abstract

**Background:**

Postoperative atrial fibrillation (POAF) commonly occurs following surgical repair of degenerative mitral regurgitation (DMR) and is associated with unfavorable outcomes. This study aimed to identify preoperative risk factors for acute POAF in patients undergoing mitral valve repair for DMR, with a specific focus on the role of preoperative echocardiography.

**Methods:**

A retrospective study was conducted involving 1127 DMR patients who underwent mitral valve repair between 2017 and 2022. The primary endpoint was the occurrence of acute POAF within 30 days after surgery. Univariate and multivariate logistic regression analyses were performed to identify risk factors for POAF. Additionally, subgroup analyses were conducted to evaluate the predictive value of preoperative parameters for the development of acute POAF.

**Results:**

Acute POAF was observed in 152 patients (13.5%). After adjusting for covariates, multivariate analysis revealed that age (odds ratio (OR) 1.05; 95% confidence interval (CI) 1.03–1.07, *p* < 0.001), hypertension (OR 1.50; 95% CI 1.03–2.21, *p* = 0.037), left ventricular ejection fraction (OR 0.95; 95% CI 0.92–0.98, *p* = 0.004), and left atrial enlargement (OR 1.03; 95% CI 1.00–1.06, *p* = 0.019) were independent predictors of acute POAF. The interventricular septum (IVS) thickness demonstrated a strong association with acute POAF (OR 1.21; 95% CI 1.06–1.38, *p* = 0.005). The optimal cut-off value for the IVS thickness in predicting acute POAF was 11.0 mm. The adjusted OR of association between an IVS thickness >11 mm and acute POAF was 1.73 (95% CI 1.03–2.89, *p* = 0.037). The IVS thickness was consistently identified as a significant predictor of POAF in the subgroup analyses.

**Conclusions:**

Preoperative assessment of clinical morbidity and echocardiographic parameters, particularly IVS thickness, can be valuable in identifying high-risk patients for acute POAF and informing targeted strategies for prevention and management.

## 1. Introduction

Degenerative mitral regurgitation (DMR) is a common valvular heart disease characterized by valvular or chordal degeneration [1]. The core mechanism of DMR is systolic excessive leaflet movement, which is defined by a prolapse in the left atrium ≥2 mm and can affect one or both leaflets and one or multiple scallops [1]. DMR continues to be a common cause of increased morbidity and mortality, and mitral valve surgery is the standard treatment for patients with symptomatic DMR. Surgical repair is generally preferred over replacement due to better outcomes and long-term survival [2]. However, postoperative complications, such as atrial fibrillation, can occur and have been associated with increased morbidity, mortality, and length of hospital stay [3, 4, 5, 6].

Postoperative atrial fibrillation (POAF) is the most common postoperative cardiac arrhythmia and has been reported to occur in up to 46.7% of patients after mitral valve surgery [7]. The pathophysiology of atrial fibrillation (AF) is complex and multifactorial, and several risk factors have been identified, including age, hypertension, diabetes mellitus, and preoperative atrial enlargement [8]. However, the risk factors for acute POAF specifically in patients with DMR who undergo surgical repair of mitral valves are not well understood. Therefore, the aim of this study is to investigate the risk factors for the occurrence of acute POAF in patients with DMR following surgical repair of mitral valves.

## 2. Methods

### 2.1 Study Participants

**Fig. 1.**
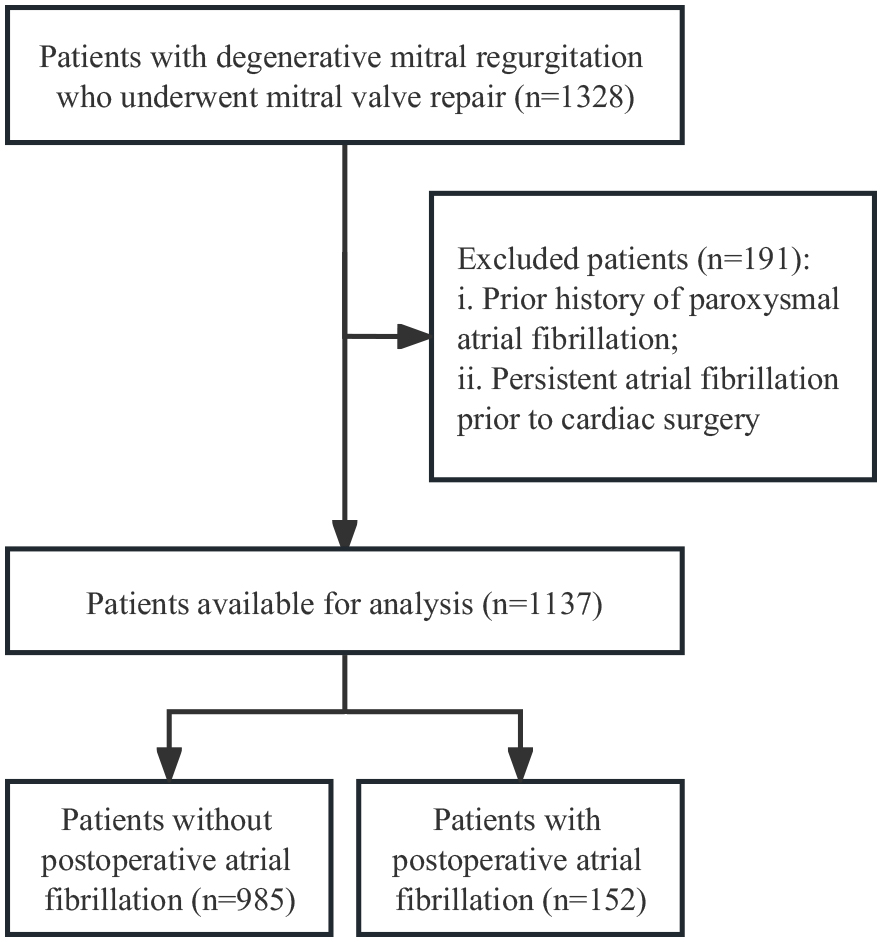
**Flow diagram of selected patients**.

This retrospective cohort study enrolled patients with DMR who underwent surgical mitral valve repair between January 1, 2017, and December 31, 2022, and fulfilled the eligibility criteria of being 18 years or older with a confirmed diagnosis of primary DMR by transthoracic echocardiography. Exclusion criteria included: patients with a prior history of atrial fibrillation defined as those who had a primary or secondary in-hospital or outpatient diagnosis of AF or prescriptions for antiarrhythmic drugs [i.e., flecainide, sotalol, amiodarone, or dronedarone] at any time prior to hospitalization for cardiac surgery, significant concomitant aortic disease, mitral stenosis, and prior valve surgery (Fig. 1). Out of the 1318 DMR patients initially screened for eligibility, 191 were excluded as they had a confirmed history of paroxysmal or persistent atrial fibrillation before cardiac surgery. A total of 1127 DMR patients who underwent mitral valve repair were included in the final analysis. The study protocol was approved by the institutional review board of our institute. Informed consents were obtained from all included patients.

### 2.2 Clinical Characteristics

Patient demographics, including age, gender, body mass index (BMI), body surface area, were collected from electronic medical records. Preoperative echocardiographic data were systematically collected to assess cardiac structure and function. These echocardiographic parameters include left atrium size, interventricular septum (IVS) thickness, posterior wall thickness, left ventricular ejection fraction (LVEF), left ventricular end-diastolic diameter, and left ventricular end-diastolic volume. Left ventricular mass index (LVMI) was calculated using a formula derived from the measurements of IVS thickness, posterior wall thickness, and left ventricular end-diastolic diameter [9]. Severity of mitral regurgitation and prolapse sites were evaluated and recorded via Doppler echocardiography. All echocardiographic studies were performed by skilled cardiac sonographers and interpreted by experienced cardiologists who were blinded to other clinical data of the patients.

### 2.3 Surgical Techniques for Mitral Valve Repair 

Patients were placed under general anesthesia and in a supine position, after which a median sternotomy was performed. Cardiopulmonary bypass was established by cannulating the ascending aorta, superior and inferior vena cava. The mitral valve was examined through a right atrial fossa ovalis or interatrial groove approach. Surgical techniques, including valve annuloplasty, valve leaflet repair, artificial chordae tendineae, chordae transfer, chordae shortening, commissuroplasty, and the edge-to-edge technique, were selected based on the examination results. A saline test was performed after the mitral valve repair, and if there was no significant regurgitation, the clamp on the ascending aorta was removed. The efficacy of the mitral valve repair and the degree of residual regurgitation were evaluated using transesophageal echocardiography. Information on concomitant procedures such as tricuspid annuloplasty, left atrium appendage closure, and coronary artery bypass graft, as well as the cardiopulmonary bypass time (CPBT) and aortic cross-clamping time (XCT), were recorded. The study included operative outcomes data, such as length of hospital stay, the length of stay in the intensive care unit, and mechanical ventilation duration, from electronic medical records. The surgical interventions were performed by experienced cardiothoracic surgeons using established methods [10].

### 2.4 Outcome Assessment

The primary endpoint of the study was to determine the incidence of acute POAF, which was defined as any episode of atrial fibrillation lasting more than 30 seconds within the first 30 days after mitral valve repair [11, 12]. In order to confirm the presence of acute POAF, electrocardiographic data obtained from medical records were reviewed by a cardiologist to ensure that the assessment was standardized. To validate the diagnosis of acute POAF, the cardiologist thoroughly examined the electrocardiogram and associated medical records to ensure accurate diagnosis. In instances where the outcome was uncertain, an independent assessment was sought from the Medical Outcome Reviewer Committee (MORC), which was comprised of two cardiologists and a cardiac electrophysiologist. The MORC conducted a comprehensive review of the electrocardiographic data and medical records to provide a final determination of the outcome. This approach ensured a standardized and objective evaluation of outcomes, thereby enhancing the reliability and validity of the study findings. The primary endpoint of this study was the occurrence of AF within 30 days postoperatively. AF detection was conducted exclusively during hospitalization, utilizing continuous telemetry and scheduled 12-lead electrocardiograms (ECGs) throughout the inpatient period. The diagnosis of POAF was established solely based on arrhythmias recorded during the hospital stay. Post-discharge, no implantable loop recorder (ILR) or ambulatory ECG monitoring was implemented.

### 2.5 Statistical Analysis

Continuous variables were presented as mean ± standard deviation or median with interquartile range, while categorical variables were presented as frequencies and percentages. Logistic regression models were employed to identify the risk factors associated with acute POAF in patients with DMR who underwent surgical repair of mitral valves. Univariable logistic regression analysis was performed to assess the association between each potential risk factor and acute POAF. Multivariate logistic regression analysis was then conducted incorporating variables that were statistically significant on univariate analysis (*p* < 0.05) or deemed clinically relevant to identify independent predictors of acute POAF. Subgroup analyses were conducted to identify potential effect modifiers of the relationship between risk factors and acute POAF. Restriction cubic splines were constructed to estimate the linear or non-linear trend associations of potential predictor and POAF risk. A receiver operating curve (ROC) analysis was performed to determine the optimal cutoff value for IVS thickness to predict acute POAF. The significance level was set at *p* < 0.05. The statistical analysis was conducted using R software version 4.1.1 (R Foundation for Statistical Computing, Vienna, Austria).

## 3. Results

### 3.1 Study Population 

A total of 1127 individuals diagnosed with DMR who underwent mitral valve repair surgery were enrolled in this study. Acute POAF occurred in 152 patients, accounting for 13.5% of the cohort. Table 1 summarizes the baseline demographics, clinical profiles, and echocardiographic findings. The average patient age was 50.7 years, with males comprising 69.7% of the study population. Hypertension was the most common comorbidity (36.6%), followed by diabetes mellitus (5.3%) and chronic obstructive pulmonary disease (COPD) (1.1%). Compared to DMR patients without POAF, those who developed acute POAF exhibited several distinctive features, including an enlarged ascending aorta (33.8 ± 4.8 mm vs. 31.9 ± 4.3 mm, *p* < 0.001), a larger left atrium (46.7 ± 7.6 vs. 44.8 ± 7.6, *p* = 0.003), and a thicker IVS (10.2 ± 1.7 vs. 9.7 ± 1.4, *p* < 0.001). Additionally, their LVEF was lower (63.7 ± 4.8 vs. 65.0 ± 5.0, *p* = 0.002).

**Table 1.** **Baseline characteristics of patients with DMR undergoing surgical repair**.

Variables		Total (n = 1127)	No POAF (n = 975)	POAF (n = 152)	*p* value
Clinical characteristics					
	Age, years	50.7 ± 12.9	49.7 ± 13.0	56.6 ± 10.8	<0.001
	Female	341 (30.3)	290 (29.7)	51 (33.6)	0.342
	BMI, kg/m^2^	24.8 ± 3.8	24.7 ± 3.7	25.4 ± 4.0	0.042
	SBP, mmHg	131.6 ± 16.8	131.3 ± 16.8	133.4 ± 16.9	0.155
	DBP, mmHg	78.6 ± 12.1	78.6 ± 12.1	78.8 ± 12.0	0.839
	NT-proBNP, pg/mL	949.2 ± 1376.3	924.2 ± 1396.6	1166.5 ± 1435.1	0.779
	NYHA Class, III–IV	278 (24.7)	240 (24.6)	38 (25)	0.919
Medical comorbidities, n (%)					
	Diabetes mellitus	60 (5.3)	50 (5.1)	10 (6.6)	0.459
	COPD	12 (1.1)	10 (1)	2 (1.3)	0.670
	Hypertension	413 (36.6)	340 (34.9)	73 (48)	0.002
Echocardiography data					
	Ascending aorta, mm	32.1 ± 4.4	31.9 ± 4.3	33.8 ± 4.8	<0.001
	Left atrium, mm	45.0 ± 7.6	44.8 ± 7.6	46.7 ± 7.6	0.003
	IVS, mm	9.8 ± 1.5	9.7 ± 1.4	10.2 ± 1.7	<0.001
	LVPW, mm	9.2 ± 0.7	9.2 ± 0.7	9.2 ± 0.8	0.145
	LVEDD, mm	57.5 ± 6.2	57.5 ± 6.1	57.8 ± 6.5	0.575
	LVEDV, mL	163.7 ± 39.2	163.8 ± 39.1	163.3 ± 40.1	0.923
	LVMI, g/m^2^	133.3 ± 30.0	132.5 ± 29.9	138.4 ± 30.4	0.026
	LVEF, %	64.9 ± 4.9	65.0 ± 5.0	63.7 ± 4.8	0.002

Abbreviations: BMI, body mass index; COPD, chronic obstructive pulmonary disease; DBP, diastolic blood pressure; DMR, degenerative mitral regurgitation; IVS, interventricular septum; LVEDD, left ventricular end-diastolic diameter; LVEDV, left ventricular end-diastolic volume; LVEF, left ventricular ejection fraction; LVMI, left ventricular mass index; LVPW, left ventricular posterior wall; NT-proBNP, N-terminal pro b-type natriuretic peptide; NYHA, New York Heart Association; POAF, postoperative atrial fibrillation; SBP, systolic blood pressure.

Regarding the operative details and outcomes (**Supplementary Table 1**), it was observed that patients with POAF were less likely to have a lesion at P1 (17.0% vs. 27.1%, *p* = 0.009). Notably, there was no difference in the proportion of concomitant surgeries or surgical techniques involved, such as artificial chords, mitral annuloplasty, or mitral ring size. Patients with acute-onset POAF had a longer duration of CPBT and mechanical ventilation, as well as a prolonged hospital stay (16.2 ± 8.1 vs. 13.6 ± 4.6 days) and intensive care unit (ICU) stay.

### 3.2 Risk Factors of Acute POAF in Patients With DMR 

Univariable logistic regression analysis (Table 2) was conducted and showed that age, hypertension, diameter of ascending aorta, left atrium size, IVS, LVEF, CPBT, duration of mechanical ventilation, length of hospital stay, and ICU stay time were significantly associated with an increased risk of POAF. Specifically, for every one-year increase in age, the odds of developing acute POAF increased by 5% (odds ratio (OR) 1.05; 95% confidence interval (CI) 1.03–1.07, *p* < 0.001). Patients with hypertension had 1.73 times higher odds of developing acute POAF (OR 1.73; 95% CI 1.22–2.44, *p* = 0.002) compared to those without hypertension. After adjusting for covariates, multivariate analysis revealed that hypertension (OR 1.50; 95% CI: 1.03–2.21, *p* = 0.037) and left atrial enlargement (OR 1.03; 95% CI: 1.00–1.06, *p* = 0.019) were independent predictors of acute POAF. IVS thickness demonstrated a strong association with acute POAF (OR 1.21; 95% CI: 1.06–1.38, *p* = 0.005) (**Supplementary Table 2**). Ventilation duration, length of hospital stay, and ICU stay were also identified as significant risk factors, with odds ratios of 1.02 (95% CI 1.01–1.03, *p* = 0.001), 1.08 (95% CI 1.05–1.11, *p* < 0.001), and 1.47 (95% CI 1.32–1.64, *p* < 0.001), respectively, per 1-unit change. Among the echocardiographic parameters assessed, IVS was found to have the highest odds ratio for acute POAF, with an OR of 1.22 (95% CI 1.09–1.37), indicating its potential usefulness as a predictor of acute POAF. Upon adjusting for age, gender, BMI, hypertension, and length of ICU stay, it was observed that IVS exhibited the strongest correlation with POAF and remained independently associated with POAF (OR 1.18, 95% 1.04–1.35, *p* = 0.012; Table 3). Through the utilization of restrictive cubic splines, a distinct linear correlation between IVS and the risk of POAF was established, exhibiting a consistent trend where an increment in IVS thickness corresponds to an elevated likelihood of POAF, even after adjusted for confounders of age, gender, BMI, hypertension and length of ICU stay (**Supplementary Fig. 1**).

**Table 2.** **Associations of clinical parameters and perioperative onset acute atrial fibrillation**.

Variables	OR	95% CI	*p* value
Age	1.05	(1.03, 1.07)	<0.001
Female	1.19	(0.83, 1.72)	0.342
Diabetes mellitus	1.30	(0.65, 2.63)	0.460
COPD	1.29	(0.28, 5.93)	0.746
Hypertension	1.73	(1.22, 2.44)	0.002
SBP	1.01	(1.00, 1.02)	0.155
DBP	1.00	(0.99, 1.02)	0.839
NT-proBNP	1.00	(1.00, 1.00)	0.771
Ascending aorta	1.11	(1.06, 1.15)	<0.001
Left atrium	1.03	(1.01, 1.05)	0.004
IVS	1.22	(1.09, 1.37)	0.001
LVPW	1.17	(0.94, 1.46)	0.149
LVEDD	1.01	(0.98, 1.04)	0.575
LVEDV	1.00	(0.99, 1.01)	0.923
LVMI	1.01	(1.00, 1.01)	0.026
LVEF	0.95	(0.92, 0.98)	0.002
Tricuspid valvuloplasty	1.10	(0.77, 1.58)	0.577
Anterior leaflet prolapse	1.10	(0.76, 1.58)	0.622
Posterior leaflet prolapse	0.88	(0.58, 1.35)	0.570
Bi-leaflet prolapse	0.91	(0.49, 1.70)	0.765
Barlow’s	0.39	(0.14, 1.09)	0.073
Artificial chord	1.26	(0.87, 1.83)	0.229
Mitral annuloplasty ring	1.48	(0.70, 3.13)	0.307
CPBT	1.01	(1.00, 1.01)	0.012
XCT	1.01	(1.00, 1.01)	0.082
Hospital stay	1.08	(1.05, 1.11)	<0.001
Ventilation duration	1.02	(1.01, 1.03)	0.001
ICU Stay	1.47	(1.32, 1.64)	<0.001

Abbreviations: CI, confidence interval; COPD, chronic obstructive pulmonary disease; CPBT, cardiopulmonary bypass time; DBP, diastolic blood pressure; ICU, intensive care unit; IVS, interventricular septum; LVEDD, left ventricular end-diastolic diameter; LVEDV, left ventricular end-diastolic volume; LVEF, left ventricular ejection fraction; LVMI, left ventricular mass index; LVPW, left ventricular posterior wall; NT-proBNP, N-terminal pro b-type natriuretic peptide; OR, odds ratio; SBP, systolic blood pressure; XCT, aortic cross-clamping time.

**Table 3.** **Multivariable analysis of echocardiographic factors associated with POAF**.

	Model 1^*^		Model 2^†^		Model 3^‡^	
Variable	OR (95% CI)	*p*	OR (95% CI)	*p*	OR (95% CI)	*p*
Left atrium	1.02 (1.00–1.05)	0.089	1.02 (1.00–1.05)	0.081	1.02 (0.99–1.04)	0.206
IVS	1.16 (1.02–1.32)	0.024	1.15 (1.01–1.31)	0.033	1.18 (1.04–1.35)	0.012
LVPW	1.12 (0.89–1.40)	0.334	1.11 (0.88–1.38)	0.380	1.13 (0.90–1.43)	0.286
LVEDD	1.02 (0.99–1.05)	0.307	1.02 (0.99–1.05)	0.258	1.01 (0.98–1.04)	0.526
LVEDV	1.00 (0.99–1.01)	0.857	1.00 (0.99–1.01)	0.565	1.00 (0.99–1.01)	0.595
LVMI	1.01 (1.00–1.01)	0.050	1.01 (1.00–1.01)	0.050	1.01 (1.00–1.01)	0.067
LVEF	0.95 (0.92–0.98)	0.005	0.95 (0.92–0.98)	0.004	0.96 (0.93–0.99)	0.018

^*^Model 1 adjusted for age, gender (female), and BMI; ^†^Model 2 adjusted for age, gender (female), BMI, and hypertension; ^‡^Model 3 adjusted for age, gender (female), BMI, and hypertension, and length of ICU stay. Abbreviations: BMI, body mass index; CI, confidence interval; ICU, intensive care unit; IVS, interventricular septum; LVEDD, left ventricular end-diastolic diameter; LVEDV, left ventricular end-diastolic volume; LVEF, left ventricular ejection fraction; LVMI, left ventricular mass index; LVPW, left ventricular posterior wall; OR, odds ratio; POAF, postoperative atrial fibrillation.

The optimal cutoff value, 11 mm, was chosen based on the point that maximized the balance between sensitivity and specificity, yielding a sensitivity of 90.1% and a specificity of 69.3%. Patients were categorized into two groups based on the optimal IVS cutoff value of 11 mm. The crude odds ratio (OR) for the association between IVS >11 mm and POAF was 1.84 (95% CI: 1.13–3.00, *p* = 0.014). After adjusting for age, gender, diabetes, and hypertension, the adjusted OR was 1.73 (95% CI: 1.03–2.89, *p* = 0.037), indicating a persistent, albeit slightly attenuated association between IVS >11 mm and acute POAF.

### 3.3 Sensitivity Analyses for IVS as a Risk Predictor of POAF

**Fig. 2.**
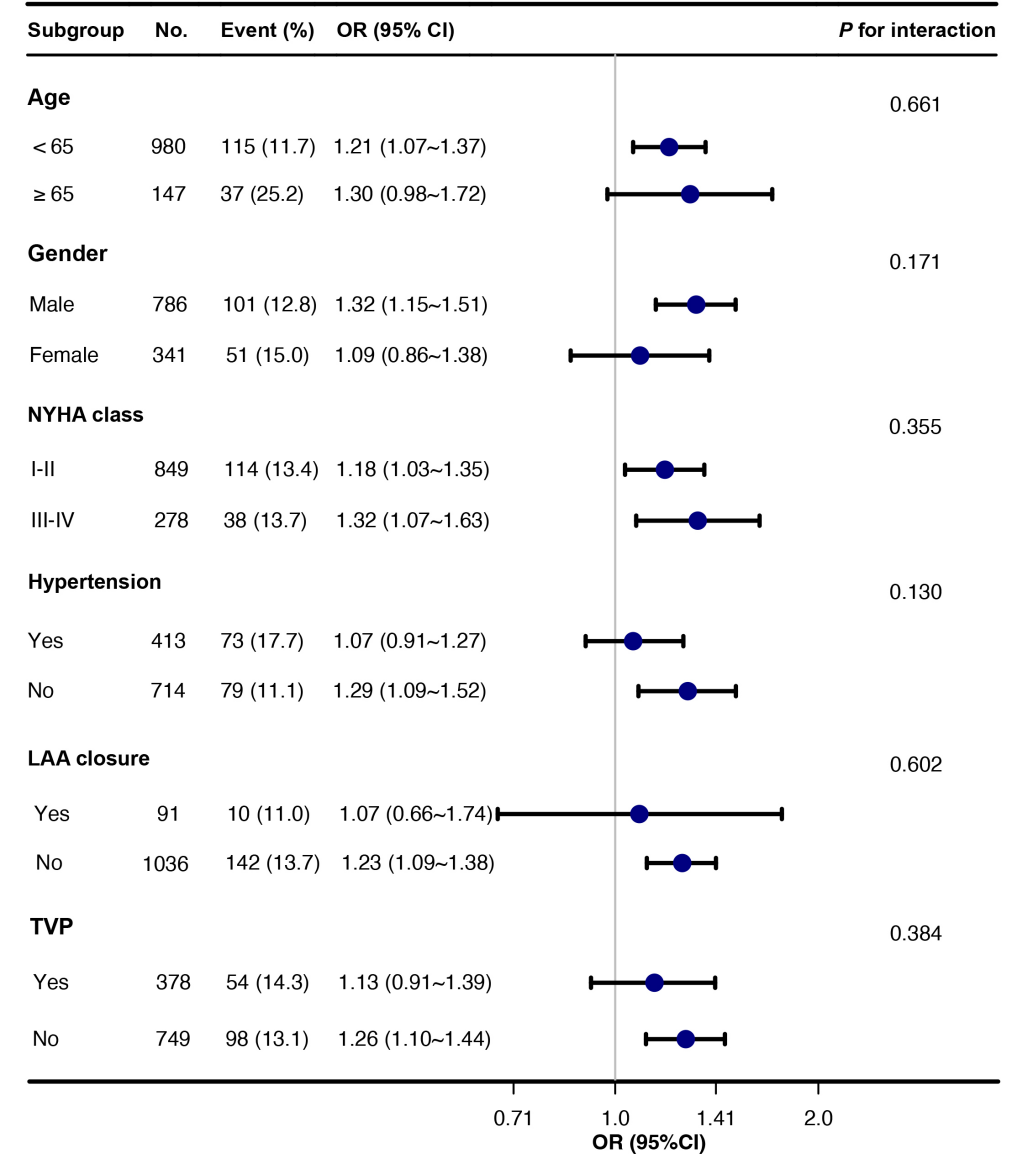
**Subgroup analyses of IVS thickness and POAF**. Abbreviations: CI, confidence interval; IVS, interventricular septum; LAA, left atrial appendage; NYHA, New York Heart Association; OR, odds ratio; POAF, postoperative atrial fibrillation; TVP, tricuspid valvuloplasty.

To explore the relationship between IVS and POAF in diverse patient populations, subgroup analyses were conducted based on age, gender, NYHA classification, hypertension, left appendage closure, and concomitant tricuspid valve repair (Fig. 2). The findings revealed a significant association between IVS and POAF, indicating that IVS consistently predicts POAF across different patient subgroups (all *p* for interaction >0.05). Sensitivity analyses were additionally performed to assess the influence of missing data and outliers on our results. Notably, these analyses yielded consistent outcomes with our primary analysis and did not impact our conclusions.

## 4. Discussion

This study has identified the occurrence and risk factors associated with acute POAF in a large population of patients undergoing mitral valve repair for DMR. Our cohort showed an incidence of POAF of 13.5%, which aligns with previously reported rates. The results indicated that advanced age, hypertension, longer length of stay in the ICU, and various echocardiographic parameters were significant risk factors for POAF in this patient population. The correlation between IVS thickness and POAF emphasizes its potential as a valuable predictor of this complication. These findings provide clinicians with valuable information to identify high-risk patients who could benefit from increased monitoring and preventive interventions to mitigate the occurrence of this commonly encountered complication after surgery. Although the absolute difference in IVS thickness between the POAF and non-POAF groups was modest (0.5 mm), this difference reached statistical significance and was consistently associated with POAF in multivariate and subgroup analyses. Moreover, a cutoff of 11 mm yielded high sensitivity (90.1%), indicating that even subtle increases in IVS thickness may be clinically meaningful when evaluated alongside other risk factors.

The present study support previous research that has identified an increased risk of POAF in patients undergoing mitral valve surgery. Our study revealed age, hypertension, and left atrial enlargement were independent risk factors for POAF, consistent with findings from other studies [3, 13, 14, 15, 16, 17]. Lee *et al*. [14] reported that age and left atrial enlargement were significant risk factors for POAF in cardiac surgery patients. Similarly, several studies [16, 17, 18] found that hypertension was a significant risk factor for POAF in cardiac surgery patients. Various risk scoring systems have been developed to predict the development of POAF. These include several risk stratification tools: the POAF score, the HATCH score—which incorporates factors such as hypertension, age, prior transient ischemic attack or stroke, chronic obstructive pulmonary disease, and heart failure—the CHA_2_DS_2_-VASc score, which evaluates congestive heart failure, hypertension, age ≥75 years (weighted double), diabetes, prior stroke or TIA (also weighted double), vascular disease, age between 65 and 74, and female sex; and the COM-AF score, which considers variables such as age, heart failure, female sex, hypertension, diabetes, and history of stroke [19, 20, 21, 22]. Among these risk scores, chronic heart failure (CHF) is consistently included as a predictive factor for POAF. However, in our study, CHF was not incorporated into the multivariate analysis, leaving it uncertain whether IVS thickness would maintain its predictive value for POAF when CHF is considered. This gap warrants further investigation to better understand the interplay between these variables. While existing scoring systems are widely utilized for POAF risk stratification, they do not comprehensively account for echocardiographic parameters such as IVS thickness. Although we did not directly compare our findings with these established risk models, our results suggest that IVS thickness, a readily accessible echocardiographic parameter, may offer incremental value in refining risk prediction.

This study encompassed a large sample size compared to previous investigations, thereby potentially enhancing the generalizability of our findings. These findings suggest that a comprehensive assessment of various preoperative clinical indicators, including interventricular septum thickness, may offer improved predictive accuracy for POAF compared to focusing solely on individual factors. Our research adds to the existing body of literature by providing additional evidence on the risk factors for POAF in patients with DMR undergoing mitral valve surgical repair. By identifying these risk factors, healthcare professionals can more effectively predict which patients are at a higher risk of developing POAF and implement appropriate preventive measures. Importantly, the results of subgroup analyses consistently demonstrated a significant association between IVS and POAF, indicating that IVS is a reliable predictor of POAF across diverse patient subgroups. This suggests that the predictive value of IVS thickness for POAF is not influenced by patient characteristics or comorbidities within these subgroups.

Numerous hypotheses have been proposed for the mechanisms underlying the observed associations between the identified risk factors and POAF. One theory suggests that age-related alterations in the autonomic nervous system may contribute to the onset of POAF by disrupting atrial electrophysiology and fostering arrhythmogenesis [23]. Additionally, modifications in the renin-angiotensin-aldosterone system and oxidative stress pathways associated with hypertension may prompt atrial remodeling and fibrosis, which, in turn, can lead to conduction anomalies and arrhythmias [24, 25]. Mechanical stress exerted on atrial tissue due to left atrial enlargement may also trigger changes in atrial electrophysiology and structural remodeling [23, 26]. The findings of this study suggest that an IVS measurement greater than 11 mm is associated with an increased risk of POAF. The adjusted odds ratio suggests that this association remains statistically significant even after accounting for potential confounding variables such as age, gender, diabetes mellitus (DM), and hypertension. These results align with previous research highlighting the potential utility of IVS measurements as a predictive marker for POAF. IVS thickness has been suggested as a marker of elevated left ventricular filling pressure [27], which may cause left atrial dilation and, consequently, POAF. Therefore, IVS thickness should be considered a significant factor when evaluating the risk of POAF in patients undergoing mitral valve repair for DMR. These mechanisms are intricate and potentially interdependent, and more extensive research is required to fully comprehend these intricate mechanisms and to identify potential targets for therapeutic intervention.

IVS thickness has been widely used as a predictor for cardiovascular events in several diseases. Park SK *et al*. [28] demonstrated that IVS thickness was associated with an elevated risk of developing hypertension among individuals without prior hypertension, with a significantly higher area under the curve (AUC) compared to left ventricular mass (LVM). Similarly, IVS thickness proved to be a valuable prognostic indicator for all-cause mortality in Chinese patients with coronary artery disease, even among those with normal LVM values [29]. Even though IVS has been identified as a regular echocardiographic parameter, its role as a predictive factor for cardiovascular events has been controversial in previous study. In the Atrial Fibrillation Follow-up Investigation of Rhythm Management (AFFIRM) Trial, patients with mildly, moderately, and severely abnormal IVS thickness had risk ratios of 2.33 (95% CI 1.34–4.06, *p* = 0.003) and 3.00 (95% CI 0.92–9.71, *p* = 0.06) for all-cause mortality over 2.5 years [30]. However, these associations were no longer statistically significant after controlling for factors such as age, sex, LVEF, presence of atrial fibrillation, valvular heart disease, and left ventricular wall motion score index. Findings from the Pressioni Arteriose Monitorate E Loro Associazioni (PAMELA) cohort indicated that IVS thickness did not remain an independent predictor of cardiovascular outcomes after adjustment for relevant clinical variables, such as the difference in risk between individuals in the lowest and highest IVS thickness quintiles [31]. Population heterogenieity, different criteria for classifying abnormal IVS thickness and choice of adjusting variants might have led to the negative results from the study [29]. The underestimated predicting value of IVS thickness should be re-evaluated from the results of more recent studies.

The identification of age, hypertension, left atrial enlargement, and interventricular septum thickness as significant risk factors for POAF in patients with DMR undergoing mitral valve repair has important clinical implications. These findings emphasize the importance of considering these risk factors when assessing the likelihood of POAF in this patient population and highlight the need for tailored strategies in risk stratification and management. For patients with multiple risk factors, more aggressive prophylactic measures, such as beta-blockers or amiodarone, may be warranted. The role of IVS thickness in predicting POAF should also be acknowledged in clinical practice, as it emerges as a novel and significant risk factor in this population. Future studies should investigate the pathophysiological processes associated with increased IVS thickness and how they contribute to atrial electrophysiological changes and arrhythmogenesis. Studies should also determine whether interventions targeting these risk factors, such as lifestyle modifications or medications, can effectively reduce the incidence of POAF in patients with DMR undergoing mitral valve repair. These findings have the potential to inform clinical decision-making and enhance outcomes for patients undergoing surgical repair of mitral valves for DMR.

In light of the findings in this study, several potential avenues for future research can be identified. One crucial area is exploring interventions aimed at modifying the identified risk factors to reduce the risk of POAF in patients with DMR undergoing mitral valve repair. Antihypertensive medications or lifestyle modifications, such as exercise and dietary changes, could be studied for their potential benefit. Further research could also be conducted to identify additional risk factors for POAF in this population, such as genetic factors or comorbidities that may contribute to atrial remodeling or electrophysiological changes. Longitudinal studies could evaluate the long-term outcomes of patients who develop POAF following mitral valve repair and determine whether this arrhythmia is associated with an increased risk of morbidity or mortality. Continued research in this area is critical for improving our understanding of the pathophysiology of POAF in this population and developing more effective strategies for the prevention and management of this common postoperative complication.

Several limitations should be considered when interpreting these results. First, due to the retrospective design, there is a risk of selection bias, which limits the ability to infer causality. Second, as the study was conducted at a single institution, the applicability of the outcomes to broader patient populations or different clinical environments may be restricted. The prediction of POAF was hindered by the unavailability of distinct measures of cardiac geometry such as left ventricular sphericity index, which may have the potential to offer valuable insights. Furthermore, the assessment of IVS thickness via echocardiography may be susceptible to measurement error or variability, potentially impacting the accuracy of the results. This limitation may have led to underestimation of the true incidence of POAF within 30 days, particularly for asymptomatic or late-onset cases occurring after discharge. Additionally, the lack of data on certain confounding factors, such as smoking or alcohol consumption, hinders the ability to fully adjust for these variables. Further research is needed to clarify the underlying mechanisms and causality between the identified risk factors and POAF, as well as to explore potential interventions for reducing the risk of POAF in patients with DMR undergoing mitral valve repair.

## 5. Conclusion

In conclusion, POAF is a common complication in patients with DMR who undergo surgical repair of mitral valves, with age, hypertension, left atrial enlargement, and IVS thickness identified as significant risk factors. Preoperative assessment of clinical morbidity and echocardiographic parameters, particularly the IVS thickness, may be beneficial in identifying patients at a high risk of POAF and in the development of targeted strategies for its prevention and management. Future research should explore whether interventions targeting these identified risk factors can reduce the incidence of POAF in this population.

## Availability of Data and Materials

The data that support the findings of this study are available from the corresponding author upon reasonable request.

## Supplementary Material

Supplementary material associated with this article can be found, in the online version, at https://doi.org/10.31083/RCM38938.
