# Supplementary material for: Preoperative Risk Factors for Acute Postoperative Atrial Fibrillation in Patients Undergoing Mitral Valve Repair for Degenerative Mitral Regurgitation: Insights Into Cardiac Geometry
Source: Rev Cardiovasc Med. 2025 Aug 29;26(8):38938. doi: 10.31083/RCM38938 (PMC12415746; doi:10.31083/RCM38938)
Supplement: Supplementary file 1 [file 2153-8174-26-8-38938-s1.zip › Supplementary Table 1.docx]

**Supplementary Table 1: Operative Characteristics and Postoperative Outcomes in Patients With and Without Acute POAF**

| **Variables** | **Total (n = 1127)** | **No POAF (n = 975)** | **POAF (n = 152)** | **P value** |
| --- | --- | --- | --- | --- |
| **Concomitant surgery** | | | | |
| **Tricuspid annuloplasty, n (%)** | 378 (33.5) | 324 (33.2) | 54 (35.5) | 0.577 |
| **LA appendix resection, n (%)** | 52 (4.6) | 44 (4.5) | 8 (5.2) | 0.682 |
| **LA appendix closure, n (%)**  **Prolapse Site** | 39 (3.5) | 37 (3.8) | 2 (1.3) | 0.12 |
| **Anterolateral commissure, n (%)** | 110 (9.8) | 93 (9.5) | 17 (11.2) | 0.525 |
| **Posteromedial commissure, n (%)** | 151 (13.4) | 130 (13.3) | 21 (13.8) | 0.871 |
| **A1, n (%)** | 144 (12.8) | 126 (12.9) | 18 (11.8) | 0.71 |
| **A2, n (%)** | 146 (13.0) | 126 (12.9) | 20 (13.2) | 0.936 |
| **A3, n (%)** | 159 (14.1) | 138 (14.2) | 21 (13.8) | 0.911 |
| **P1, n (%)** | 290 (25.7) | 264 (27.1) | 26 (17.1) | 0.009 |
| **P2, n (%)** | 539 (47.8) | 470 (48.2) | 69 (45.4) | 0.519 |
| **P3, n (%)** | 357 (31.7) | 299 (30.7) | 58 (38.2) | 0.065 |
| **Artificial chord, n (%)** | 296 (26.3) | 250 (25.6) | 46 (30.3) | 0.228 |
| **Annuloplasty rings** |  |  |  |  |
| Mitral Ring, n (%) | 1045 (92.7) | 901 (92.4) | 144 (94.7) | 0.304 |
| Mitral Ring Size, n (%) | 989(87.8) | 851(87.3) | 138(90.8) | 0.467 |
| **26** | 4 (0.4) | 3 (0.3) | 1 (0.7) |  |
| **27** | 6 (0.5) | 5 (0.5) | 1 (0.7) |  |
| **28** | 102 (9.1) | 81 (8.3) | 21 (13.8) |  |
| **29** | 31 (2.8) | 28 (2.9) | 3(2) |  |
| **30** | 316 (28.0) | 272 (27.9) | 44 (28.9) |  |
| **31** | 81 (7.2) | 72 (7.4) | 9 (5.9) |  |
| **32** | 331 (29.4) | 287 (29.4) | 44 (28.9) |  |
| **33** | 7 (0.6) | 5 (0.5) | 2 (1.3) |  |
| **34** | 94 (8.3) | 82 (8.4) | 12 (7.9) |  |
| **36** | 16 (1.4) | 15 (1.5) | 1 (0.7) |  |
| **38** | 1 (0.1) | 1 (0.1) | 0 (0) |  |
| **Tricuspid Ring, n (%)** | 351 (31.1) | 301 (30.9) | 50 (32.9) | 0.69 |
| **Cardiopulmonary bypass time (minutes), Mean ± SD** | 105.8 ± 43.6 | 104.3 ± 41.0 | 115.1 ± 56.9 | 0.011 |
| **Aortic cross clamp time (minutes), Mean ± SD** | 75.0 ± 31.9 | 74.3 ± 31.2 | 79.7 ± 35.8 | 0.081 |

Abbreviations: BMI = Body Mass Index; LA = left atrial; LV = left ventricular
