# Supplementary material for: Preoperative Risk Factors for Acute Postoperative Atrial Fibrillation in Patients Undergoing Mitral Valve Repair for Degenerative Mitral Regurgitation: Insights Into Cardiac Geometry
Source: Rev Cardiovasc Med. 2025 Aug 29;26(8):38938. doi: 10.31083/RCM38938 (PMC12415746; doi:10.31083/RCM38938)
Supplement: Supplementary file 1 [file 2153-8174-26-8-38938-s1.zip › Supplementary Figure 1 .pdf]

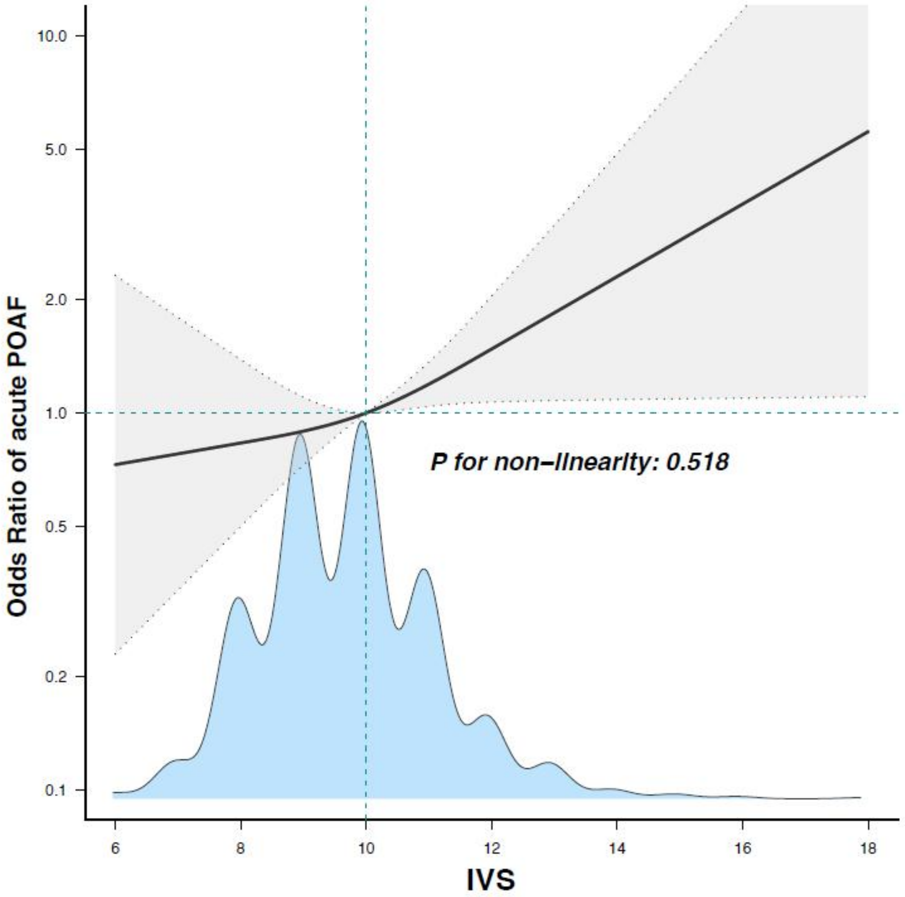

Supplementary **Figure 1. Association between IVS and adjusted Risk of POAF by restrictive cubic splines.** The associations were adjusted for age, gender (female), BMI, hypertension and length of ICU stay. BMI, body mass index; ICU, intensive care unit; IVS, interventricular septum; POAF, postoperative atrial fibrillation
