# Supplementary material for: Preoperative Risk Factors for Acute Postoperative Atrial Fibrillation in Patients Undergoing Mitral Valve Repair for Degenerative Mitral Regurgitation: Insights Into Cardiac Geometry
Source: Rev Cardiovasc Med. 2025 Aug 29;26(8):38938. doi: 10.31083/RCM38938 (PMC12415746; doi:10.31083/RCM38938)
Supplement: Supplementary file 1 [file 2153-8174-26-8-38938-s1.zip › Supplementary Table 2.docx]

| **Supplementary Table 2** — Associations of clinical parameters and perioperative onset atrial fibrillation after adjusting for covariates | | | | | | | | | |
| --- | --- | --- | --- | --- | --- | --- | --- | --- | --- |
| Variables | Unadjusted | | | Model 1^†^ | | | Model 2^‡^ | | |
|  | OR | 95% CI | *P* | OR | 95% CI | *P* | OR | 95% CI | *P* |
| Age | 1.05 | (1.03, 1.07) | <0.001 | 1.05 | (1.03, 1.07) | <0.001 | 1.05 | (1.03, 1.07) | <0.001 |
| Hypertension | 1.73 | (1.22, 2.44) | 0.002 | 1.65 | (1.14, 2.37) | 0.007 | 1.50 | (1.03, 2.21) | 0.037 |
| ICU Stay | 1.47 | (1.32, 1.64) | <0.001 | 1.47 | (1.32, 1.64) | <0.001 | 1.47 | (1.31, 1.65) | <0.001 |
| Ascending Aorta Diameter | 1.11 | (1.06, 1.15) | <0.001 | 1.10 | (1.06, 1.15) | <0.001 | 1.10 | (1.06, 1.15) | <0.001 |
| LA | 1.03 | (1.01, 1.05) | 0.004 | 1.03 | (1.01, 1.05) | 0.011 | 1.03 | (1.00, 1.06) | 0.019 |
| LVEF | 0.95 | (0.92, 0.98) | 0.002 | 0.95 | (0.92, 0.98) | 0.003 | 0.95 | (0.92, 0.98) | 0.004 |
| IVS | 1.22 | (1.09, 1.37) | 0.001 | 1.22 | (1.08,1.38) | 0.002 | 1.21 | (1.06,1.38) | 0.005 |
| LVM | 1.00 | (1.00, 1.01) | 0.027 | 1.00 | (1.00, 1.01) | 0.029 | 1.00 | (1.00, 1.01) | 0.048 |
| LVMI | 1.01 | (1.00, 1.01) | 0.025 | 1.01 | (1.00, 1.01) | 0.015 | 1.01 | (1.00, 1.01) | 0.034 |

| ^*^Each line represents a separate model. ^†^Model 1 adjusted for gender, NYHA, BMI and DM. ^‡^Model 2 adjusted for gender, NYHA, BMI, DM, left atrial appendage operation and mitral ring size. NYHA, New York Heart Association functional classification; BMI, body mass index; DM, diabetes milicus. |
| --- |
